# Giant natal cleft epidermal inclusion cyst surgically excised in a young male in Australia

**DOI:** 10.1093/jscr/rjaf141

**Published:** 2025-03-18

**Authors:** William Layt, Brielle Williams, Richard Maguire

**Affiliations:** Department of General Surgery, Gold Coast Hospital and Health Service, 1 Hospital Blvd, Southport, QLD 4215, Australia; Department of General Surgery, Gold Coast Hospital and Health Service, 1 Hospital Blvd, Southport, QLD 4215, Australia; Department of General Surgery, Gold Coast Hospital and Health Service, 1 Hospital Blvd, Southport, QLD 4215, Australia

**Keywords:** giant, epidermal inclusion cyst, surgical excision, natal cleft, pilonidal

## Abstract

Epidermal inclusion cysts are benign lesions commonly found in hair-bearing areas. Giant epidermal inclusion cysts (GEICs), those exceeding 5 cm in diameter, are rare and may pose diagnostic challenges, especially in atypical locations. We report the case of a 29-year-old male with a 7–8 cm swelling in the natal cleft, diagnosed as a GEIC after imaging studies, including ultrasound, CT, and MRI. Surgical excision was performed with careful dissection to preserve surrounding structures. The patient had an uneventful recovery. This case highlights the diagnostic and surgical challenges of GEICs in the natal cleft and emphasizes the importance of pre-operative imaging and meticulous surgical technique to avoid complications.

## Introduction

Epidermal inclusion cysts (EICs) are common, benign, congenital tumours located in the subcutaneous plane. These are typically slow-growing lesions that can arise due to trauma [1, 2]. These cysts generally develop from the distal part of the hair follicle or within the sebaceous gland. As such, they are typically found in hair-bearing areas like the face, scalp, neck, and trunk. Less common sites include the palms, soles, penis, and buttocks [1].

Whilst EICs typically measure 1–5 cm in diameter, GEICs exceed 5 cm in diameter and have a rarer occurrence [3, 4]. These lesions can be challenging to differentiate from other soft-tissue conditions, including pilonidal disease, lipomas, dermoid cysts, neoplasms, and skin carcinomas [5]. To assist in pre-operative planning, ultrasound and MRI are considered gold-standard and assist in ruling out intramuscular involvement (although rare) [6].

Here, we present the case of a young male who presented with a significant swelling in the natal cleft, suspicious of a GEIC.

## Case report

A 29-year-old male was referred to the colorectal surgery outpatient department clinic at a tertiary hospital by their general practitioner. He had a swelling in the natal cleft that had been present for ~2 years, with worsening pain over this time. This swelling doubled in size in the 3-month period before presenting to the surgical outpatient department. He denied any history of trauma, discharge, bowel symptoms, or systemic symptoms. His only past medical history was asthma, for which he takes a salbutamol puffer. The only surgical history he had was a perineal/scrotal abscess incision and drainage ~2 years prior.

**Figure 1:**
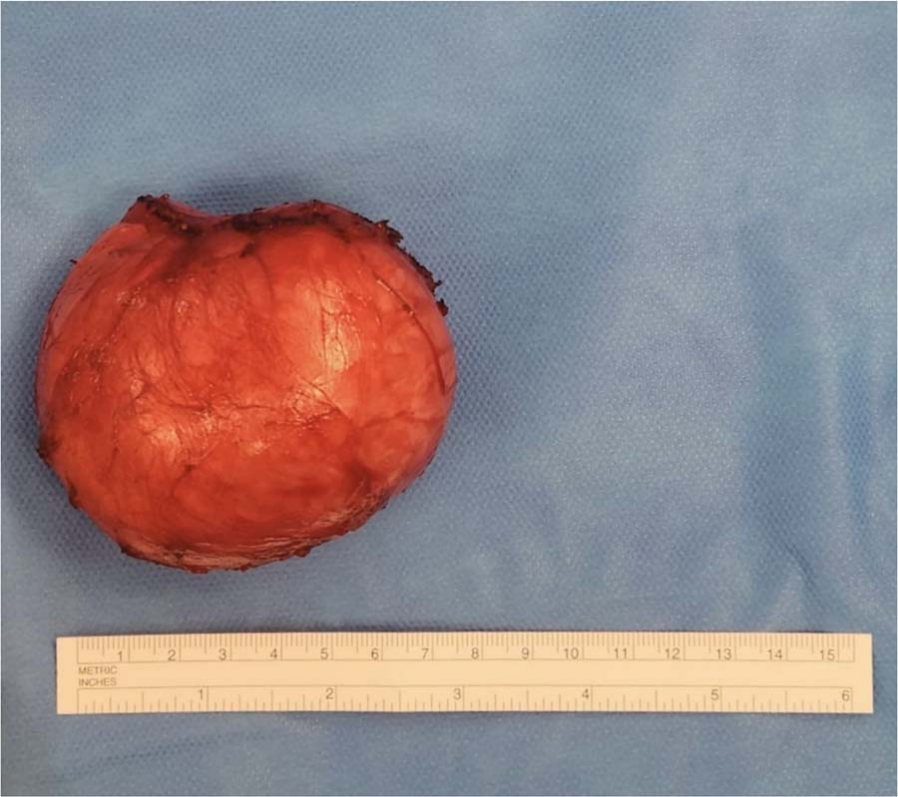
Excised EIC from natal cleft measuring 7 cm in diameter.

On examination, there was a sizeable fluctuant mass in the midline of the natal cleft extending to the superior edge of the anus, measuring 7–8 cm in length (Fig. 1). It was non-tender with no surrounding erythema or discharge.

**Figure 2:**
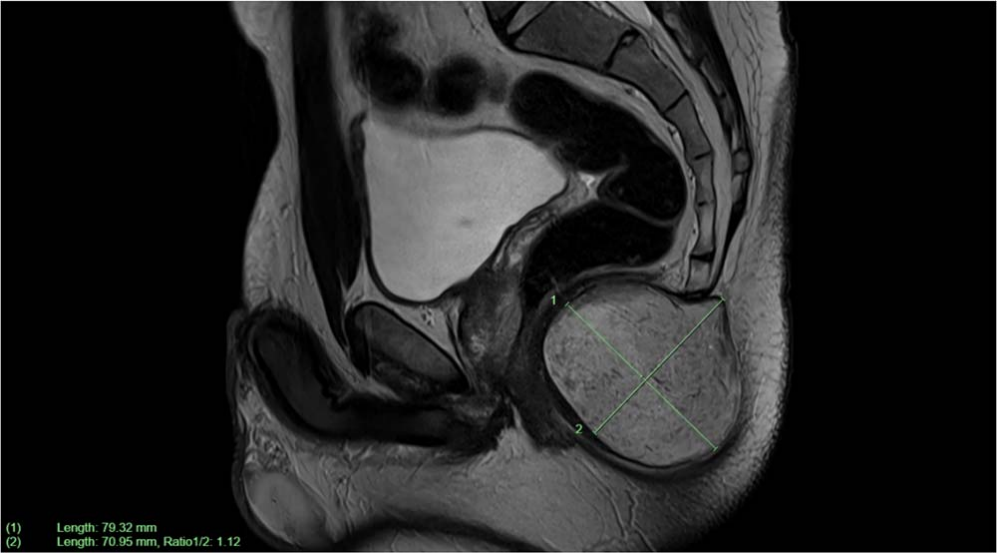
Sagittal plane MRI image of large subcutaneous cystic structure compressing on the anal canal—measuring 7.9 × 7.1 cm in size.

The GP had initially organized an ultrasound of the lesion, which was reported to have a solid hypoechoic inhomogeneous focus in the natal cleft. Subsequently, a CT pelvis scan showed a well-circumscribed low-density lesion at the distal coccyx. An MRI was then completed for surgical planning, showing a subcutaneous perineal mass causing elevation of the levator ani muscle and anterior indentation of the anal canal. There was no evidence of a fistula, abscess, infection, or an aggressive neoplastic process (Fig. 2). The initial preliminary diagnosis of this lesion was a tailgut cyst or EIC, and the patient was subsequently booked for an excision of said cyst.

Performed under general anaesthetic, the procedure involved making a transverse incision over the cyst away from the anal verge. Whilst keeping the cyst capsule intact, circumferential dissection was completed to free the cyst from surrounding structures (Fig. 1). These structures included the posterior wall of the rectum and the sphincter complex, preserving both. Part of the periosteum of the coccyx was also dissected to maintain the plane of dissection.

The patient remained in hospital for 1-day post-surgery and was discharged without concerns. The drain remained for 1-week post-surgery and was removed in the outpatient general surgical department.

## Discussion

This case discusses the rare presentation of a GEIC in the natal cleft. Not commonly seen in the natal cleft, GEICs are typically seen in anatomical areas with abundant sebaceous glands and pose a diagnostic challenge in these rarer locations. This case explores the diagnostic approach and subsequent surgical excision of this lesion.

The natal cleft is predisposed to conditions like pilonidal disease, often complicating the initial diagnosis. In order to differentiate GEICs from other soft tissue conditions, imaging with ultrasound and MRI must be employed [6]. These imaging modalities also play an important role in surgical planning. Histopathological examination remains the gold standard for confirming the diagnosis and ruling out malignancy, particularly in atypical presentations or recurrent cases [7, 8].

Surgical excision is the preferred treatment for GEICs, with complete removal of the cyst and its lining critical to preventing recurrence [6]. This can be challenging in the natal cleft due to the risk of wound dehiscence and delayed healing in an area prone to friction and moisture. In this case, meticulous dissection, primary closure, and postoperative care minimized complications [1, 6].

As a benefit of Australia's highly accessible healthcare system, early diagnosis and management are essential in improving patient outcomes, as highlighted by this case. Due to the rarity of GEICs in the natal cleft, clinicians must consider a wide range of differential diagnoses for soft tissue masses in unusual locations.

This report contributes to the limited literature on GEICs in the natal cleft, offering insights into diagnostic and surgical excision approaches. Future research focusing on long-term outcomes and recurrence rates could further guide best practices in managing these lesions surgically and non-surgically.

### Learning points

GEICs are uncommon in the natal cleft and require careful differentiation from conditions like pilonidal disease.

Preoperative imaging and meticulous excision are essential to ensure complete removal and prevent recurrence.

Appropriate wound management is critical for successful healing in high-friction areas like the natal cleft.
